# Identification of *Brucella* by MALDI-TOF Mass Spectrometry. Fast and Reliable Identification from Agar Plates and Blood Cultures

**DOI:** 10.1371/journal.pone.0014235

**Published:** 2010-12-06

**Authors:** Laura Ferreira, Silvia Vega Castaño, Fernando Sánchez-Juanes, Sandra González-Cabrero, Fabiola Menegotto, Antonio Orduña-Domingo, José Manuel González-Buitrago, Juan Luis Muñoz-Bellido

**Affiliations:** 1 Unidad de Investigación, Hospital Universitario de Salamanca, Salamanca, Spain; 2 Departamento de Microbiología, Hospital Universitario de Salamanca, Salamanca, Spain; 3 Departamento de Anatomía Patológica, Microbiología, Medicina Preventiva y Salud Pública, Medicina Legal y Forense, Universidad de Valladolid, Valladolid, Spain; 4 Departamento de Bioquímica y Biología Molecular, Universidad de Salamanca, Salamanca, Spain; 5 Departamento de Medicina Preventiva, Salud Pública y Microbiología Médica, Universidad de Salamanca, Salamanca, Spain; Universidad Nacional, Costa Rica

## Abstract

**Background:**

MALDI-TOF mass spectrometry (MS) is a reliable method for bacteria identification. Some databases used for this purpose lack reference profiles for *Brucella* species, which is still an important pathogen in wide areas around the world. We report the creation of profiles for MALDI-TOF Biotyper 2.0 database (Bruker Daltonics, Germany) and their usefulness for identifying brucellae from culture plates and blood cultures.

**Methodology/Principal Findings:**

We created MALDI Biotyper 2.0 profiles for type strains belonging to *B. melitensis* biotypes 1, 2 and 3; *B. abortus* biotypes 1, 2, 5 and 9; *B. suis*, *B. canis*, *B ceti* and *B. pinnipedialis*. Then, 131 clinical isolates grown on plate cultures were used in triplicate to check identification. Identification at genus level was always correct, although in most cases the three replicates reported different identification at species level. Simulated blood cultures were performed with type strains belonging to the main human pathogenic species (*B. melitensis*, *B. abortus*, *B. suis* and *B. canis*), and studied by MALDI-TOF MS in triplicate. Identification at genus level was always correct.

**Conclusions/Significance:**

MALDI-TOF MS is reliable for *Brucella* identification to the genus level from culture plates and directly from blood culture bottles.

## Introduction

Brucellosis is a zoonosis that remains an important public health problem in wide areas, such as the Mediterranean basin, the north of Africa, Mexico, and Central and South America [1]–[4]. Six species have been described, based on host preferences, metabolism, culture and antigenic features, including the two most recent species (*Brucella pinnipedialis* and *B. ceti*), isolated from seals and cetaceans (dolphins and whales), but most human cases remain to be caused by *B. melitensis* and *B. abortus* [5]. However, DNA-DNA hybridization shows a high homology between strains, indicating that current species should be rather considered as subspecies corresponding to evolutionary lineages adapted to specific hosts [6].

Classically, biphasic blood cultures such as the Ruiz-Castañeda method were used to isolate brucellae from blood and bone marrow. Now, most laboratories use continuous-monitoring automated blood culture systems, which can shorten the time to isolation and have been shown to be highly sensitive [7]. Nevertheless, subculture is necessary to identify the microorganism, and brucellae may require 2–3 days to grow on chocolate or blood agar. Rapid automated bacterial identification systems must be interpreted with caution, because brucellae have been misidentified with some of these systems [8]. PCR have shown high sensitivity and specificity, but its use remains infrequent, mainly due to standardization problems [9].

MALDI-TOF mass spectrometry (MS) has been suggested as a fast and reliable method for bacterial identification [10], [11], based on protein profiles characteristic of each microorganism. Databases have been developed that include the main pathogenic microorganisms, thus allowing the use of this method in routine bacterial identification from plate culture. Nevertheless, *Brucella* has not been still incorporated to some of the main databases available, because of problems derived from their potential bioterrorist use. This is an important problem for the routine use of MALDI-TOF MS for the direct diagnosis of blood cultures in countries where brucellosis is still frequent.

The aim of our study was to identify and differentiate *Brucella* species by MALDI-TOF MS, combining MALDI-TOF MS with dedicated bioinformatics and statistical methods (database search and pattern-matching algorithm). Initial spectra from three type strains of *B. melitensis*, five type strains of *B. abortus* and one type strain of *B. suis*, *B. canis*, *B. ceti* and *B. pinnipedialis* were used to set up database entries for re-identification of *Brucella* strains. This database was evaluated with 131 blind-coded *Brucella* clinical isolates previously identified by conventional methods. We also tested the reliability of this method for identifying brucellae directly from blood cultures, as soon as artificially inoculated blood cultures were reported as positive by a continuous-monitoring automated blood culture system.

## Materials and Methods

### Ethic Statement

Sheep blood was used for some experiments, i.e. simulated blood cultures. Since Sheep blood is obtained, as a conventional laboratory product, from commercial sources (Pronadisa Conda, Madrid, Spain), we did not consider any ethics approval to be necessary for this study.

### Microorganisms

The strains used for generating reference spectra were the following: *B. melitensis* biotype 1, strain 16M (ATCC 23456); *B. melitensis* biotype 2, strain 63/9 (ATCC 23457); *B. melitensis* biotype 3, strain ETHER (ATCC 23458); *B. abortus* biotype 1, strain 544 (ATCC 23448); *B. abortus* biotype 1, strain 45/20 (NCTC 11361); *B. abortus* biotype 2, strain 86/8/59 (ATCC 23449); *B. abortus* biotype 5, strain B3196 (ATCC 23452); *B. abortus* biotype 9, strain C68 (ATCC 23455), *B. suis* (NCTC 10098), *B. canis* (NCTC 10854), *B. ceti* (NCTC 12891) and *B. pinnipedialis* (NCTC 12890) Microorganisms were plated onto chocolate agar plates (bioMérieux, France), and incubated at 37°C for 48 hours. Colonies were used for creating Biotyper 2.0 database profiles.

The same isolates were also spread onto blood agar plates (bioMérieux, France), under the same conditions, to check the score reported by MALDI-TOF for colonies obtained from different culture media.

One hundred and thirty one human clinical isolates were used as blind coded isolates to check the reliability of the Biotyper 2.0 database, once spectra for *B. melitensis*, *B. abortus*, *B. canis*, *B. ceti*, *B. pinnipedialis* and *B. suis* had been created. The clinical isolates were plated onto chocolate agar plates (bioMérieux, France), and incubated at 37°C for 48 hours. Then, colonies were identified by conventional microbiology methods and PCR, according previously described methods [12], and by MALDI-TOF MS.

### Colonies samples preparation for MALDI-TOF MS

Cells of a whole colony were transferred from the plate to a 1.5 mL tube (Eppendorf, Germany) with a pipette tip and mixed thoroughly in 300 µL of water to resuspend the bacterial cells. Then, 900 µL of absolute ethanol was added and the mixture was centrifuged at 15,500 *g* for 2 min and the supernatant was discarded. The pellet was air-dried at room temperature for 1 hour. Subsequently, 50 µL of formic acid (70% v/v) were added to the pellet and mixed thoroughly by pipetting before the addition of 50 µL of acetonitrile to the mixture. The mixture was centrifuged again at 15,500 *g* for 2 min. One microliter of the supernatant was placed onto a spot of the steel target and air-dried at room temperature. Each sample was overlaid with 1 µL of matrix solution (saturated solution of HCCA (alpha-cyano-4-hydroxy cinnamic acid) in organic solvent (50% acetonitrile and 2.5% trifluoroacetic acid) and air-dried.

#### Simulated blood cultures

The same 3 *B. melitensis*, 5 *B. abortus*, 1 *B. canis* and 1 *B. suis* type strains used for establishing reference spectra, were used to perform simulated blood cultures. Three to four colonies were suspended into sterile water to reach a concentration around 10^4^ CFU/mL. Then, 4 mL of brucellae suspension were added to 4 mL of blood, and these 8 mL were inoculated into BACTEC Plus + Aerobic/F bottles (Becton Dickinson, NJ, USA), and incubated at 37°C in a BACTEC 9240 device (Becton Dickinson, NJ, USA), until they were reported as positive.

### Blood cultures samples preparation for MALDI-TOF MS

Four mL of the positive blood culture were centrifuged at 2,000 *g* for 30 seconds to remove leucocytes. Supernatant was centrifuged at 15,500 *g* for 5 minutes to collect bacteria. The pellet was washed once with de-ionized water. Then, the ethanol/formic acid extraction procedure described above was applied.

### MALDI-TOF MS

Measurements were performed on an Autoflex III MALDI-TOF/TOF mass spectrometer (Bruker Daltonics, Leipzig, Germany) equipped with a 200-Hz smartbeam laser. Spectra were recorded in the linear positive mode at a laser frequency of 200 Hz within a mass range from 2,000 to 20,000 Da. The IS1 voltage was 20 kV, the IS2 voltage was maintained at 18.6 kV, the lens voltage was 6 kV, and the extraction delay time was 40 ns.

For each spectrum, 500 laser shots were collected and analyzed (10×50 laser shots from different positions of the target spot). The spectra were calibrated externally using the standard calibrant mixture (*Escherichia coli* extracts including the additional proteins RNase A and myoglobin, Bruker Daltonics). Calibration masses were as follows: RL36, 4364.3 Da; RS22, 5095.8 Da; RL34, 5380.4 Da; RL33meth, 6254.4 Da, RL32, 6315 Da; RL29, 7273.5 Da; RS19, 10299.1 Da; RNase A, 13682.2 Da; myoglobin, 16952.5 Da.

### Spectrum generation and data analysis

For automated data analysis, raw spectra were processed using the MALDI Biotyper 2.0 software (Bruker Daltonics, Leipzig, Germany) at default settings. The software performs normalization, smoothing, baseline subtraction, and peak picking, creating a list of the most significant peaks of the spectrum (*m/z* values with a given intensity, with the threshold set to a minimum of 1% of the highest peak and a maximum of 100 peaks). To identify unknown bacteria, each peak list generated was matched directly against reference libraries (3,476 species) using the integrated patterns matching algorithm of the Biotyper 2.0 software (Bruker Daltonics, GmbH, Germany). The unknown spectra were compared with a library of reference spectra based on a pattern recognition algorithm using peak position, peak intensity distributions and peak frequencies. Once a spectrum has been generated and captured by the software, the whole identification process is performed automatically, without any user intervention. MALDI-TOF identifications were classified using modified score values proposed by the manufacturer: a score ≥2 indicated species identification; a score between 1.7 and 1.9 indicated genus identification, and a score <1.7 indicated no identification.

For reference library construction, 36 independent spectra were recorded for each bacterial isolate (three independent measurements at twelve different spots each).

Manual and visual estimation of the mass spectra was performed using Flex Analysis 3.0 (Bruker Daltonics GmbH, Germany) performing smoothing and baseline substraction. Checking existence of flatlines, outliers or single spectra with remarkable peaks differing from the other spectra was done, taking into account that mass deviation within the spectra set should be less than 500 ppm. Finally, 20 spectra were selected, removing questionable spectra from the collection. To create peak lists of the spectra, the BioTyper software was used as described above. The 70 independent peaks of a strain were used for automated “main spectrum” generation with default settings of the BioTyper software. Thereby, for each library entry a reference peak list (main spectrum) which contains information about averaged masses, averaged intensities, and relative abundances in the 20 measurements for all characteristic peaks of a given strain was created, so a main spectrum displayed the most reproducible peaks typical for a certain bacterial strain.

Cluster analysis was performed based on comparison of strain-specific main spectra created as described above. The dendrogram was constructed by the statistical toolbox of Matlab 7.1 (MathWorks Inc., USA) integrated in the MALDI Biotyper 2.0 software. The parameter settings were: ‘Distance Measure = Correlation’ and ‘Linkage = average’. The linkage function is normalized according to the distance between 0 (perfect match) and 1000 (no match).

## Results

**Figure 1:**
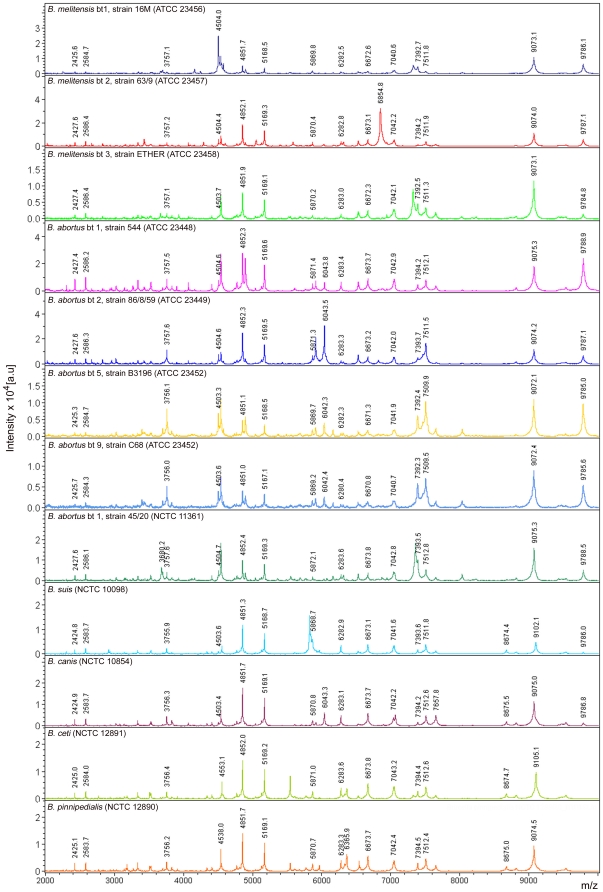
MALDI-TOF MS spectra of whole-cell extracts of *Brucella* reference strains in the range from 2000 to 11000 Da. The relative intensity of the ions (a.u., arbitrary units) are shown on the *y* axis, and the masses (in Da) of the ions are shown on the *x* axis. The *m/z* value stands for mass to charge ratio. For a single positive charge, this value corresponds to the molecular weight of the protein.

MALDI Biotyper 2.0 database (Bruker Daltonics) used for routine identification of microorganism in clinical microbiology contains 3,476 entries, involving bacteria and fungi groups. Although it contains a wide variety of clinically relevant microorganisms, some important pathogenic microorganisms have not been yet included (e.g. *Brucella*). This is an important limitation in some countries, where brucellosis is still a relevant infectious problem and it is not infrequent to isolate *Brucella* species from febrile patients. For this reason, 3 *B. melitensis*, 5 *B. abortus*, 1 *B. suis*, 1 *B. canis*, 1 *B. ceti* and 1 *B. pinnipedialis* type strains were used to generate reference spectra and to extend the MALDI Biotyper database. The visual inspection of these spectra from whole-cell extracts revealed a high similarity among them (Figure 1), with specific peaks commons for all the strains at 2426 Da, 2585 Da, 3757 Da, 4851 Da, 5168 Da, 5870 Da, 6282 Da, 6672 Da, 7042 Da, 7393 Da, and 7511 Da (Table 1). Although some differences can be observed among all the strains, only some strains have discriminating peaks at species level. Thus, peaks at 4244, 4286 and 6854 Da appear only in *B. melitensis*, but none of these peaks are constant in all the *B. melitensis* strains. The same happens with peaks at 3899, 6166 and 7358 Da in *B. abortus*. According these data, there is a peak profile characteristic of *Brucella* at genus level, but there isn't at species level.

**Table 1:** Discriminating peaks of *Brucella* strains and biotypes analyzed by MALDI-TOF MS.

	peaks (m/z)																
	2426	2585	2671	2827	3337	3680	3757	3899	4068	4159	4244	4286	4338	4410	4504	4538	4851
*B. mellitensis* bt. 1, strain 16M (ATCC 23456)	x	x			x		x			x	x			x	x	x	x
*B. mellitensis* bt. 2, strain 63/9 (ATCC 23457)	x	x	x	x	x		x		x	x		x		x	x	x	x
*B. mellitensis* bt. 3, strain ETHER (ATCC 23458)	x	x	x	x	x		x		x						x	x	x
*B. abortus* bt. 1, strain 544 (ATCC 23448)	x	x	x	x	x		x	x	x					x	x	x	x
*B. abortus* bt. 2, strain 86/8/59 (ATCC 23449)	x	x	x	x	x		x	x	x					x	x	x	x
*B. abortus* bt. 5, strain B 3196 (ATCC 23452)	x	x			x		x							x	x	x	x
*B. abortus* bt. 9, strain C68 (ATCC 23455)	x	x					x								x	x	x
*B. abortus* bt.1, strain 45/20 (NCTC 11361)	x	x			x	x	x		x	x				x	x	x	x
*B. suis* (NCTC 10098)	x	x			x		x						x	x	x		x
*B. canis* (NCTC 10854)	x	x			x		x		x				x	x	x	x	x
*B. ceti* (NCTC 12891)	x	x			x		x						x	x			x
*B. pinnipedialis* (NCTC 12890)	x	x			x		x						x	x		x	x

	peaks (m/z)																
	5168	5870	5918	6042	6166	6282	6672	6854	7042	7327	7358	7393	7511	8675	9074	9103	9787
*B. mellitensis* bt. 1, strain 16M (ATCC 23456)	x	x				x	x		x	x		x	x		x		x
*B. mellitensis* bt. 2, strain 63/9 (ATCC 23457)	x	x				x	x	x	x			x	x		x		x
*B. mellitensis* bt. 3, strain ETHER (ATCC 23458)	x	x				x	x		x	x		x	x		x		x
*B. abortus* bt. 1, strain 544 (ATCC 23448)	x	x	x	x		x	x		x			x	x		x		x
*B. abortus* bt. 2, strain 86/8/59 (ATCC 23449)	x	x	x	x		x	x		x			x	x		x		x
*B. abortus* bt. 5, strain B 3196 (ATCC 23452)	x	x	x	x	x	x	x		x			x	x		x		x
*B. abortus* bt. 9, strain C68 (ATCC 23455)	x	x	x	x	x	x	x		x			x	x		x		x
*B. abortus* bt.1, strain 45/20 (NCTC 11361)	x	x			x	x	x		x		x	x	x		x		x
*B. suis* (NCTC 10098)	x	x				x	x		x			x	x	x		x	x
*B. canis* (NCTC 10854)	x	x	x	x		x	x		x			x	x	x	x		x
*B. ceti* (NCTC 12891)	x	x				x	x		x	x		x	x	x		x	
*B. pinnipedialis* (NCTC 12890)	x	x				x	x		x			x	x	x	x		

The m/z value stands for mass to charge ratio. For a single positive charge, this value corresponds to the molecular weight of the protein. The mass tolerance is considered ±2 Da for each peak.

**Figure 2:**
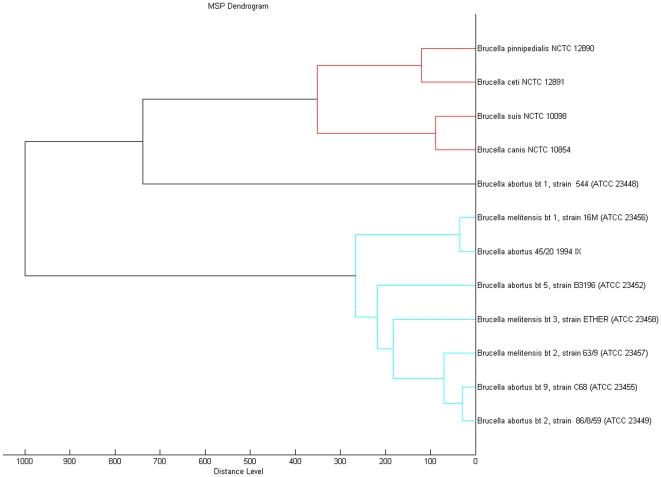
Cluster analysis of MALDI-TOF MS spectra of *Brucella* strains and biotypes. Distance is displayed in relative units.

A cluster analysis with the 12 type strains was performed, using the integrated tools of the MALDI Biotyper 2.0 software package. The resulting dendrogram (Figure 2) shows a close proximity between *B. ceti and B. pinnipedialis*, and between *B. suis* and *B. canis*, and more proximity between these four species than between any of them and *B. melitensis* and *B. abortus*. Otherwise, *B. melitensis* and *B. abortus* biotypes are extremely close and interrelated, excepting *B. abortus* biotype 1 strain 544 (ATCC 23448).

In addition, we tested the influence of different culture conditions. Therefore the different strains were cultivated on blood agar and chocolate agar and comparison between identification and scores obtained did not reveal any significant difference between both culture media in any aspect (data not shown).

Once established the spectra for the *Brucella* reference strains, 131 blind-coded *Brucella* strains were matched against the MALDI Biotyper 2.0 database in triplicate, to prove its suitability for routine identification and discrimination of *Brucella* at the genus and species levels (Table 2). The MALDI-TOF device reported the same identification at species level for the three replicates in 14 out of 17 *B. abortus* isolates (82.4%), but in only 12 out of 112 *B. melitensis* isolates (10.7%) and in 1 out of 2 *B. suis* isolates (50%). Therefore only 20.6% isolates reported the same identification at species level for the three replicates. Nevertheless, in all of them this identification was coincident with conventional and genetic identifications at genus level. In all the clinical isolates tested, the score difference between the ten most probably matched species reported by the MALDI Biotyper software for each isolate was very low and higher than 2 in several of them. As an example, we show in Table 3 the score difference between the ten most probably matched species reported for one *B. melitensis* clinical isolate. Taking into account that a reliable identification at species level is accepted with scores higher than 2, we can't discriminate between different *Brucella* species, since different *Brucella* species give scores higher than 2 in most clinical isolates.

**Table 2:** Identification by MALDI-TOF mass spectrometry and conventional identification of 131 blind-coded *Brucella*.

Conventional Identification (n° isolates)	MALDI-TOF Identification				
	Correlation at the genus level (%)	Correlation at the species level (%)			
		3/3^*^	2/3^*^	1/3^*^	0/3^*^
***B. abortus*** ** (17)**	100	82.4	11.8	0	5.9
***B. melitensis*** ** (112)**	100	10.7	8.9	23.2	57.1
***B. suis*** ** (2)**	100	50	0	0	50
Total (131)	100	20.6	9.2	19.8	50.4

Each strain was spotted three times (replicates 1, 2 and 3).

• **No. of replicates.**

**Table 3:** Ten best matches for each replicate of a blind-coded clinical isolate.

	Replicate 1		Replicate 2		Replicate 3	
	Matched Pattern	Score	Matched Pattern	Score	Matched Pattern	Score
		Value		Value		Value
1	*Brucella melitensis* bt 1, strain 16M (ATCC 23456)	2.293	*Brucella abortus* bt 9, strain C68 (ATCC 23455)	2.229	*Brucella melitensis* bt 1, strain 16M (ATCC 23456)	2.204
2	*Brucella abortus* bt 2, strain 86/8/59 (ATCC 23449)	2.161	*Brucella melitensis* bt 1, strain 16M (ATCC 23456)	2.204	*Brucella melitensis* bt 2, strain 63/9 (ATCC 23457)	2.11
3	*Brucella abortus* bt 1, strain 544 (ATCC 23448)	2.158	*Brucella abortus* bt 5, strain B3196 (ATCC 23452)	2.204	*Brucella abortus* bt 2, strain 86/8/59 (ATCC 23449)	2.092
4	*Brucella abortus* bt 9, strain C68 (ATCC 23455)	2.148	*Brucella abortus* bt 1, strain 544 (ATCC 23448)	2.166	*Brucella abortus* bt 1, strain 544 (ATCC 23448)	2.087
5	*Brucella abortus* bt 5, strain B3196 (ATCC 23452)	2.138	*Brucella abortus* bt 2, strain 86/8/59 (ATCC 23449)	2.083	*Brucella abortus* bt 5, strain B3196 (ATCC 23452)	2.064
6	*Brucella abortus* 45/20 (NCTC 11361)	2.125	*Brucella melitensis* bt 2, strain 63/9 (ATCC 23457)	2.043	*Brucella abortus* 45/20 (NCTC 11361)	2.034
7	*Brucella melitensis* bt 2, strain 63/9 (ATCC 23457)	2.124	*Brucella abortus* 45/20 (NCTC 11361)	1.985	*Brucella abortus* bt 9, strain C68 (ATCC 23455)	2.006
8	*Brucella canis* (NCTC 10854)	2.062	*Brucella suis* (NCTC 10098)	1.908	*Brucella suis* (NCTC 10098)	1.898
9	*Brucella suis* (NCTC 10098)	2.056	*Brucella canis* (NCTC 10854)	1.902	*Brucella canis* (NCTC 10854)	1.867
10	*Brucella melitensis* bt 3, strain ETHER (ATCC 23458)	1.919	*Brucella ceti* (NCTC 12891)	1.847	*Brucella melitensis* bt 3, strain ETHER (ATCC 23458)	1.743

This isolate was identified as *B. melitensis* by conventional and genetic methods.

To test the reliability of MALDI-TOF MS for identifying brucellae directly from blood culture, we inoculated blood culture bottles with type strains belonging to the four main human pathogenic species (*B. melitensis*, *B. abortus*, *B. suis* and *B. canis*). All the blood cultures inoculated were reported as positive by 48 hours of incubation. Three replicates of each positive blood culture were studied using MALDI-TOF MS. All the blood cultures were reported by the MALDI-TOF as *Brucella*. Nevertheless, in several cases the identification did not match with the organism inoculated in the blood culture at the species level, or different identifications were obtained at the species level among the three replicates of the same blood culture (Table 4).

**Table 4:** MALDI Biotyper 2.0-based identification of blood cultures spiked with different *Brucella* species and biotypes.

	Organism reported by MALDI-TOF (best match)					
Strains inoculated in blood cultures	Replicate 1	Score value	Replicate 2	Score value	Replicate 3	Score value
*B. abortus* bt. 1, strain 45/20 (NCTC 11361)	*B. melitensis* bt. 1, strain 16M (ATCC 23456)	2.09	*B. abortus* 45/20 1994 IX	2.16	*B. abortus* 45/20 1994 IX	2.125
*B.abortus* bt. 5, strain B3196 (ATCC 23452)	*B. canis* (NCTC 10854)	2.319	*B. abortus* bt. 1, strain 544 (ATCC 23448)	2.376	*B. abortus* bt. 1, strain 544 (ATCC 23448)	2.256
*B. abortus bt. 2, strain 86/8/59 (ATCC 23449)*	*B. abortus* bt. 1, strain 544 (ATCC 23448)	2.329	*B. canis* (NCTC 10854)	2.068	*B. abortus* bt. 9, strain C68 (ATCC 23455)	2.216
*B. abortus* bt. 9, strain C68 (ATCC 23455)	*B. abortus* bt. 1, strain 544 (ATCC 23448)	2.268	*B. abortus* bt. 1, strain 544 (ATCC 23448)	2.321	*B. canis* (NCTC 10854)	2.252
*B. melitensis* bt. 1, strain 16M (ATCC 23456)	*B. melitensis* bt. 1, strain 16M (ATCC 23456)	2.219	*B. melitensis* bt. 1, strain 16M (ATCC 23456)	2.224	*B. melitensis* bt. 1, strain 16M (ATCC 23456)	2.213
*B. melitensis* bt. 2, strain 63/9 (ATCC 23457)	*B. melitensis* bt. 1, strain 16M (ATCC 23456)	2.121	*B. abortus* bt. 1, strain 544 (ATCC 23448)	2.216	*B. canis* (NCTC 10854)	2.224
*B. abortus* bt. 1, strain 544 (ATCC 23448)	*B. abortus* bt. 1, strain 544 (ATCC 23448)	2.388	*B. abortus* bt. 1, strain 544 (ATCC 23448)	2.257	*B. abortus* bt. 1, strain 544 (ATCC 23448)	2.355
*B. melitensis* bt. 3, strain ETHER (ATCC 23458)	*B. canis* (NCTC 10854)	1.888	*B. melitensis* bt 3, strain ETHER (ATCC 23458)	1.943	*B. canis* (NCTC 10854)	1.995
*B. suis* (NCTC 10098)	*B. suis* (NCTC 10098)	2.465	*B. suis* (NCTC 10098)	2.429	*B. suis* (NCTC 10098)	2.481
*B. canis* (NCTC 10854)	*B. canis* (NCTC 10854)	1.929	*B. canis* (NCTC 10854)	2.007	*B. canis* (NCTC 10854)	1.983

## Discussion

Brucellosis remains a serious problem in wide areas around the world. Symptoms are nonspecific (fever, malaise, back pain, profuse night sweating). This lack of specificity of symptoms may delay the diagnosis for weeks. Though mortality rate is currently low, it remains a severe disease, and complications such as epididymo-orchitis, arthritis (especially sacroiliitis), and CNS complications are not infrequent. The current taxonomy of this genus is confusing because traditional, phenotype-based classification, and genetic-based classification are used simultaneously [5]. Genetic taxonomic studies have suggested that the 8 currently accepted species, including the 2 marine mammal-associated species, represent a single species [6], [13]. Nevertheless, recent studies show that traditional species, and the marine mammals-associated species, can be differentiated by outer membrane genes polymorphism, insertion sequences and whole-chromosome preparations [5], [14].

Human brucellosis has been described associated to *B. melitensis, B. abortus*, *B. suis* and *B. canis*, among the traditional species. Human cases associated to the newer marine mammals-associated species have also been reported [15], [16]. Though *B. abortus* has the broadest geographical distribution, most human cases are now caused by *B. melitensis*, which usually produces the most severe disease.

Microbiological procedures for *Brucella* detection and identification have been controversial for years. Blood, and eventually bone marrow, are usually the specimens for diagnosis of human brucellosis. Conventional blood cultures did not yield satisfactory results, and this led to the development of the classic biphasic blood cultures technique proposed by Ruiz-Castañeda. Now, both lyses-centrifugation methods and continuous monitoring blood cultures devices are able to detect *Brucella* with a good sensitivity [7].

Early suspicion that an isolate might be a *Brucella* is important, both for clinical and epidemiological reasons, because of the hazard of laboratory-acquired brucellosis, and for the early detection of a hypothetical bioterrorist attack. Early suspicion may be currently established based on Gram staining, but final identification requires colonies growth on agar plates and performing biochemical or serological tests on these colonies.

For all those reasons, the availability of methods that allow a rapid and reliable *Brucella* identification both from agar plates and directly from the blood culture bottles, once this is reported as positive by the continuous-monitoring blood culture systems, would be extremely useful. Direct detection methods based on PCR have been described [17], but these methods are expensive, can be affected by PCR inhibitors present in blood, and do not give any information about the viability of the microorganisms. Thus, they have not reached a wide diffusion by now.

MALDI-TOF MS is an important and increasingly available tool in clinical microbiology laboratories, because it allows a rapid and accurate identification of bacteria [10], [11]. We have seen that MALDI-TOF MS allows a fast and highly reliable identification of *Brucella*, at genus level, from colonies growth. Nevertheless, identification at species level is less reliable. Data in Figure 1 and Table 1 show a common profile for all *Brucella* type strains tested, but no specific peaks are found in any species. This explains the reliability at genus level and the low reliability at species level. Otherwise, the high similarity between all type strains is not surprising, since genetic taxonomic studies suggest that the 6 currently accepted species represent a single species [6], [13]. Once the protein profiles for *B. melitensis*, *B. abortus*, *B. suis*, *B. canis*, *B. ceti* and *B. pinnipedialis* had been created and included in the database, all the blind-coded *Brucella* isolates tested were correctly identified, with scores >2. Scores differences between the 10 most probably matched species given by the MALDI Biotyper software for each isolate were extremely low, and replicates of the same protein extract were sometimes identified as different species. In almost all isolates, more than one species matched with score value >2 in the 10 most probably matched species, thus confirming the low discriminating power at species level. The agar on which the microorganism grows does not seem to affect to identification, since scores obtained from blood agar and chocolate agar were similar for all the isolates tested. Previous studies on other microorganisms agree with these results, showing that MALDI-TOF MS protein fingerprints are not significantly influenced by variability in growth conditions [18]–[20].

MALDI-TOF MS has been previously shown to be able to identify microorganisms directly from blood cultures reported as positive by automatic blood culture processing devices [21]–[25]. In our study, positive blood cultures spiked with brucellae were reported always as *Brucella*, but species failures were frequent, as had been shown previously in agar plates. *Brucella* was always identified with scores >2 but, as happens in agar cultures, score differences between the ten most probably matched species reported by the MALDI Biotyper software for each isolate were very low, and species identification had scores >2 in the best and second match, although the species in both cases are different, indicating that the species identification cannot be fully reliable.

Protein profiles found for type strains, show that protein profile similarity does not correlate always with the traditional genus/species/biotype classification. Proteins profile for some species and biotypes may be closer to other species than to other biotypes belonging to the same species, as can be observed in cluster analysis (Figure 2).

In summary, the protein profiles for type strains of *B. melitensis* biotypes 1, 2 and 3; *B. abortus* biotypes 1, 2, 5 and 9; *B. suis*; *B. canis*; *B. ceti* and *B. pinnipedialis* were generated and included in the MALDI Biotyper database. The study of clinical isolates both from agar plate cultures and directly from blood cultures showed that these profiles allow a reliable identification of brucellae to genus level, stating that MALDI-TOF MS is an fast and highly reliable technique for straightforward *Brucella* identification, both from culture plates and directly from blood culture vials.
